# Challenges with routine data sources for PMTCT programme monitoring in East Africa: insights from Tanzania

**DOI:** 10.3402/gha.v8.29987

**Published:** 2015-12-28

**Authors:** Annabelle Gourlay, Alison Wringe, Jim Todd, Denna Michael, Georges Reniers, Mark Urassa, Prosper Njau, Deborah Kajoka, Levina Lema, Basia Zaba

**Affiliations:** 1 Faculty of Epidemiology and Population Health, London School of Hygiene and Tropical Medicine, London, United Kingdom; 2 National Institute for Medical Research, Mwanza, Tanzania; 3 National Program for Prevention of Mother-to-Child Transmission of HIV, Ministry of Health and Social Welfare, Dar Es Salaam, Tanzania

**Keywords:** HIV, Africa, data collection, maternal health services, monitoring, evaluation, implementation research methods

## Abstract

Routinely collected clinic data have the potential to provide much needed information on the uptake of services to prevent mother-to-child transmission (PMTCT) of HIV, and to measure HIV prevalence in pregnant women. This article describes the methodological challenges associated with using such data, based on the experiences of researchers and programme implementers in Tanzania and drawing from other examples from East Africa. PMTCT data are routinely collected in maternal and child health (MCH) clinics in East Africa using paper-based registers corresponding to distinct services within the PMTCT service continuum. This format has inherent limitations with respect to maintaining and accurately recording unique identifiers that can link patients across the different clinics (antenatal, delivery, child), and also poses challenges when compiling aggregate data. Recent improvements to recording systems include assigning unique identifiers to HIV-positive pregnant women in MCH clinics, although this should ideally be extended to all pregnant women, and recording mother and infant identifiers alongside each other in registers. The use of ‘health passports’, as in Malawi, which maintains the same antenatal clinic identifier over time, also holds promise. Routine data hold tremendous potential for clinic-level patient management, surveillance, and evaluating PMTCT/MCH programmes. Linking clinic data to community research datasets can also provide population-level estimates of coverage with PMTCT services, currently a problematic but vital statistic for monitoring programme performance and negotiating donor funding. Enhancements to indexing and recording of routine PMTCT/MCH data are needed if we are to capitalise on this rich data source.

Prevention of mother-to-child transmission (PMTCT) HIV services have recently come under the spotlight, with the United Nations (UN) Global Plan and Millennium Development Goals outlining ambitious targets for the elimination of new paediatric HIV infections and for improvements in maternal and child health (MCH) by 2015 (1, 2). Despite considerable progress – a reduction of 60% in new HIV infections in children globally between 2001 and 2013 – an alarming number of infections continue to take place: 240,000 estimated in 2013 (3). Africa bears the brunt of the epidemic with over 90% of paediatric infections occurring in the region (3).

PMTCT programmes comprise a cascade of services including provider-initiated HIV testing at antenatal clinics (ANC) and labour wards, provision of antiretroviral (ARV) drugs [prophylaxis or lifelong antiretroviral treatment (ART)] to HIV-positive pregnant women and their infants, delivery in a health facility, infant HIV testing, and long-term HIV care. Collection of high-quality routine data on these services and outcomes for HIV-positive mothers and HIV-exposed infants is not only essential for monitoring and evaluation of PMTCT programmes relative to local, national, and international targets, but also paramount in the clinical management of patients and in managing stocks of HIV test kits and drugs. Recording and using data from each service component is important, as one component may suggest high coverage and impact, but may mask dropouts further along the cascade (4).

Linkage of PMTCT clinic data to community-level research data such as demographic surveillance systems (DSS) (10 sites in Africa with HIV sero-surveillance) (5, 6) could provide direct estimates of coverage with PMTCT services; currently a problematic statistic. Recent UNAIDS estimates for several African countries are unrealistic, with coverage reaching over 95% (3). This suggests over-reporting of the number of pregnant women receiving ARVs (the numerator, based on aggregate statistics using routine clinic records), or inaccuracies in estimating the number of pregnant HIV-positive women (the denominator). Inaccuracies in calculating this core indicator can have profound implications, including for negotiating donor funding for PMTCT programmes, monitoring progress towards internationally agreed targets, identifying weaknesses in PMTCT programmes, and subsequently implementing necessary interventions to bring about improvements in coverage. As such, improvements in the accuracy of routine data and additional methods for direct measures of PMTCT coverage are urgently needed.

There is an added urgency to improving the quality of routine PMTCT programme data, as many African countries are currently considering abandoning ANC sentinel surveillance in favour of using HIV prevalence estimates based on routine PMTCT statistics, as the numerical basis for the latter is much larger and the geographical coverage much wider. This topic is currently under discussion in the UNAIDS reference groups on surveillance, and the reference group on estimates, projections, and modelling (7).

This paper describes some of the challenges associated with collecting and using routine PMTCT programme data, based on the practical experiences of researchers and PMTCT programme managers in Tanzania, with the ultimate goal of recommending ways to enhance these systems and realise the potential of this data source. It is hoped the experiences from Tanzania and comparisons with data collection systems in MCH clinics in other East African countries will provide useful insights for PMTCT programme implementation and management in the wider region.

## Methods

The information and recommendations presented are based primarily on observations made while collecting data from ANC, PMTCT, and HIV care and treatment clinics (CTC) for a research project to describe uptake of PMTCT services in a rural Tanzanian community. Full details of the data collection methods are described elsewhere (6). In brief, data were abstracted from routine ANC and PMTCT clinic logbooks from four health facilities in Kisesa, north-western Tanzania, and from the computerised CTC database at Kisesa health centre. Data collection was carried out in 2012, with retrospective inclusion of records back to 2005. This project also entailed the linkage of clinic records to community cohort (DSS) records from the same geographic area. Notes were made as logbooks were prepared for data entry, as the analysis was conducted, and following informal discussions with ANC nurses and CTC doctors during the data collection period and subsequent visits to the field site. Some of the findings also stem from in-depth interviews with health workers and officials, conducted as part of a qualitative study to identify barriers to PMTCT service use in Kisesa in 2012 (8, 9). Feedback from researchers working at other DSS sites in East Africa and from Tanzanian PMTCT programme managers was also incorporated. Comparisons to other settings were also made through review of the literature on routine data.

## Challenges with routine data collection

### Split location of PMTCT service provision and integration with MCH services

PMTCT data are routinely collected in MCH clinics in Africa, reflecting integration of the PMTCT programme into these services. The degree of integration between MCH and HIV care and treatment services varies, with some MCH and HIV clinics located on the same site, and other stand-alone ANCs referring HIV-positive patients to HIV clinics elsewhere. Infant blood samples are usually collected on-site, but are often tested for HIV at larger hospitals that house the necessary laboratory equipment. The spectrum of PMTCT services may thus encompass several physical locations within one facility, or incorporate different facilities, adding to the complexity of data capture and linkage throughout the service continuum.

### Multiple registers and volume of paperwork

The collection of routine PMTCT data in many African clinics takes place using various paper-based registers, each covering a different service step. Taking Tanzania and Malawi as examples, HIV test results, ARV drugs dispensed during pregnancy, ARVs during delivery, and infant HIV test results are recorded across three or four different registers. In some contexts, including Tanzania, these registers are used alongside the standard suite of MCH registers (e.g. general pregnancy register that records patient names without HIV test results). This constitutes a large volume of paperwork, with duplicated information, and a consequent burden on staff workload. Frequent updates to PMTCT registers, primarily to accommodate rapidly changing guidelines, have also resulted in multiple versions of each register, placing further demands on staff to learn and adopt new practices, as well as the need for regular training. Similar challenges with the multiplicity of registers have also been identified as an issue in PMTCT programmes in southern Africa (10).

### Lack of unique identifiers (IDs)

The ability to track outcomes of women and infants through the entire PMTCT cascade is contingent on linking records held in each PMTCT register, and on linking maternal and infant records. This necessitates a unique ID for each woman and infant. However, current systems for assigning IDs can give rise to duplicates, presenting considerable challenges when attempting to link records and monitor programme adherence and retention, and reducing the accuracy of reported statistics (e.g. the numbers of women accessing ARVs). The lack of unique IDs within MCH/PMTCT services is not an issue confined to Tanzania, having been noted in Kenya and Malawi (11).

In Tanzania and Malawi, ANC numbers are assigned to pregnant women on their first ANC visit, but each facility uses the same numbering system, giving rise to duplicate IDs between clinics. Switching facilities is fairly common (partly related to service accessibility, migration and, for HIV-positive women, potentially stigma), particularly for delivery, and it is then difficult to distinguish between patients from different facilities assigned the same ANC number (duplicate IDs). Equally problematic is the identification of women who are assigned a new ANC number when they change clinics, and thus appear on two distinct registers with different IDs (multiple IDs). The ANC numbering system is used to identify patients within the PMTCT programme, with no specific PMTCT IDs assigned. Tanzanian PMTCT registers do not contain patient names for confidentiality reasons, so the ANC number is the only means of linking patient records held in different PMTCT registers within the same facility. The ability to monitor patients’ clinical progress and attendance at each PMTCT service is therefore compromised by the lack of unique ID, with the potential for mismatching records. Duplicate and multiple IDs also complicate the linkage of ANC and HIV clinic records, or maternal and infant PMTCT records (historically a weakness of PMTCT data, although commendable improvements have been made in Tanzania to capture both the infant and mother's IDs in one register), and the aggregation of data at a national level. Similar issues have been reported in Kenya, with double counting of statistics sometimes arising as a result of repeat visits to ANC that are documented on a new line of the register (12).

### Poor data quality and health systems issues

General data quality issues, such as missing data (ANC numbers, follow-up visits, entire rows missing, loss of logbooks, and torn pages) and duplicate records, also limit the accuracy of results based on routinely collected data. Such issues have also been reported in other settings, for instance South Africa (13). Although poor data quality occasionally reflects the supply of resources (e.g. new logbooks), it is likely that health workers’ limited engagement with the data for their own planning, monitoring, or research purposes is the primary explanation for poorly completed and stored records. Insufficient training of health workers who are responsible for recording the information may be a further reason, while lack of auditing at a facility level means that poorly completed records are not identified and dealt with. These concerns point to the broader health systems issues affecting data quality, faced by many countries in this region. For example, staff shortages, with few staff stretched across various services, mean record-keeping is sometimes compromised in the effort to ensure coverage of services to all patients. Limited infrastructure, including transport and accessibility issues (particularly for rural clinics), inadequate clinic storage facilities, the heavy reliance on paper-based records, and finite supplies of materials for record completion, also play a role.

## Recommendations and conclusions

### Assigning unique IDs

Ideally, pregnant women and their infants should each be assigned a unique ID on enrolment into the PMTCT programme. Tanzanian HIV care and treatment clinics have already implemented a unique numbering system for patients, based on area and facility codes where the person first registers for care, plus serial number (for example, of the format 50-10-0500-000001 where the first two digits represent the region, the second two digits represent the district, the fifth to eighth digits represent the facility, and the final six digits are the patient number). This unique ID is maintained when patients change facility; documented on transfer forms and patient-held cards. A similar system is currently being implemented in Tanzanian MCH services for HIV-infected pregnant or breastfeeding women as new guidelines are rolled out [Option B+, lifelong ART for all pregnant women (14)], although it might theoretically be used for all pregnant women. Ideally, each clinic would be issued a list of unique numbers by a central office, to be allocated to patients upon registration.

### Enhancements to recording and storage systems

Although investments in establishing electronic medical records in MCH services would clearly facilitate data storage and usage, the costs and infrastructure required would be prohibitive in many African countries. Useful enhancements can be made to paper-based systems: the patient's unique ANC/PMTCT number would be recorded in each register by each clinic, as well as on the woman's ANC card with the dates of attendance at each PMTCT service, and on the infant's under-5 card. Issuing a booklet to women documenting multiple pregnancies, similar to the ‘health passport’ used in Malawi, and maintaining the same ANC number would avoid double counting women in programme coverage statistics and aid clinical management by making patients’ pregnancy histories more accessible to health workers. Use of filing systems with patient records filed by the number of the health passport, with clinical information updated at each visit, would also facilitate cross-service links for each patient and could aid the production of summary reports.

Streamlining PMTCT registers into fewer books, where services take place in close proximity, would facilitate follow-up and reduce paperwork for health workers. In facilities where statistics are compiled manually for reporting to higher levels, distinguishing *first* receipt of each service (e.g. first ANC visit per pregnancy, or first positive HIV test) would avoid double counting (e.g. when patients switch clinics) without the need to search on ID. This would also, importantly, improve the accuracy of compiled data and indicators for self-assessment at individual facilities.

### Health worker training

Health workers should be trained about the importance of recording the ANC number, or tracing it from earlier ANC records with the aid of the registration date if the woman returns without her ANC card (a common reason for missing ANC numbers), and motivated to take ownership of the data for their own monitoring purposes. The importance of bringing ANC cards to all follow-up visits, including delivery and child clinics, should be emphasised to pregnant women and their relatives. Although increasing the number of health workers may be unrealistic in the near future, additional training in how to complete and file registers (particularly when registers are updated), providing further mentorship and supervision by senior staff, as well as training in auditing records could lead to improvements in the shorter term.

### Improve linkage between ANC, CTC, and infant records

To further strengthen the linkage between ANC and HIV clinic records, CTC IDs must be accurately recorded by nurses in all PMTCT registers, and CTC clinicians and data entry clerks should be trained to record the ANC number of pregnant HIV-positive patients in HIV clinic records (rarely documented in the available field in Tanzania). It is also important to capture HIV-exposed infant IDs alongside mother IDs, as well as infant prophylaxis and HIV testing results: useful additions that have recently been made to the Tanzanian CTC database.

## Conclusions

Strengthening the indexing and recording of routine PMTCT data would not only capitalise on this rich data source for service monitoring and patient management at a facility level, but would facilitate more accurate estimates of PMTCT programme coverage at a national level, and reduce the burden for health workers. Improving PMTCT data will necessitate investments in health systems (e.g. staffing, training, health infrastructure) and enhancements to MCH data more broadly, although attention to the latter would also benefit the clinical management of all pregnant women and infants, and monitoring of MCH service use. We must ensure that data monitoring systems keep pace with rapidly evolving guidelines and advances in PMTCT service delivery.
